# Preoperative dental screening can reduce periprosthetic infections of hip and knee endoprostheses in the first month after surgery: results of a cohort study

**DOI:** 10.1007/s15010-023-02128-2

**Published:** 2023-12-07

**Authors:** Fabian Fenske, Benjamin Kujat, Leah Krause, Stephan Meyer, Anna-Katharina Sander, Jaqueline Repmann, Michael Neuhaus, Rainer Haak, Andreas Roth, Bernd Lethaus, Dirk Ziebolz, Gerhard Schmalz

**Affiliations:** 1 Department of Oral and Maxillofacial Surgery, University of Leipzig, Liebigstr. 12, 04103 Leipzig, Germany; 2 Specialized Clinic for Orthopedics, Mediclin Waldkrankenhaus Bad Düben, 04848 Bad Düben, Germany; 3 Department of Cariology, Endodontology and Periodontology, University of Leipzig, 04103 Leipzig, Germany; 4 Department of Orthopaedics, Trauma and Plastic Surgery, University Hospital Leipzig, 04103 Leipzig, Germany

**Keywords:** Periprosthetic joint infection, Oral health, Oral focus, Prevention

## Abstract

**Purpose:**

The oral cavity and, in particular, potential oral foci might pose a risk of periprosthetic joint infection (PJI). The aim of this cohort study was to determine whether practical preoperative dental screening would reduce the prevalence of early PJI in the first month after surgery.

**Methods:**

Patients attending a specialized endoprosthesis implantation clinic between 2018 and 2022 were recruited. Two groups were examined. The test group consisted of patients attending the clinic between 2020 and 2022 and who were referred to their family dentist using a standardized form. The comparison group consisted of patients who were treated in the clinic between 2018 and 2020. They were not referred to their family dentist. The two groups were compared for the prevalence of PJI. Univariate analysis followed by multiple logistic regression was performed to confirm risk factors for PJI in this cohort.

**Results:**

2560 individuals (test group: 1227, comparison group: 1333) were included. The prevalence of PJI was significantly lower in the test group (0.8% vs. 1.8%, *p* = 0.04). Multiple logistic regression with PJI as the dependent variable showed that a dental referral was a strong predictor of a lower prevalence of PJI (OR: 0.43, CI_95_ 0.205–0.917, *p* = 0.03). Male gender was also strongly associated with a higher frequency of PJI (OR: 2.68, CI_95_ 1.32–5.42, *p* = 0.01). Age (OR: 1.06, CI_95_ 1.01–1.10, *p* = 0.01) and BMI (OR: 1.11, CI_95_ 1.05–1.17, *p* < 0.01) had little effect on the risk of PJI.

**Conclusion:**

Dental referral using a standardized form can reduce the prevalence of early PJI. Accordingly, orthopedists and dentists should collaborate in this practical way.

**Supplementary Information:**

The online version contains supplementary material available at 10.1007/s15010-023-02128-2.

## Introduction

Rising life expectancy and the consequences of demographic change are potentially leading to an increased prevalence of age-related diseases, such as arthritic joint degeneration. Consequently, the number of joint replacements is increasing, as an analysis of economic, medical, and population data of OECD countries from 2015 has shown [1]. Though the implantation and insertion of endoprostheses has remarkable postoperative success in terms of pain reduction, restoration of physical mobility, and improvement of quality of life, postoperative complications create enormous challenges [2–5]. In particular, periprosthetic joint infection (PJI), with a perioperative, hematogenous, or per continuitatem route of infection, has a far-reaching effect from medical and economic points of view [6]. After all, PJI therapy involves 2–4 weeks of intravenous, followed by several weeks of oral antibiotic medication as well as surgical debridement or even removal of the endoprosthesis [7]. However, the consequences not only are substantial for the patient from a medical perspective, but also mean an enormous additional financial expense for the health system [8].

While the key to avoiding postoperative complications (such as aseptic loosening and peri-prosthetic fractures) is constant optimization of the implant technique, the risk of a PJI can be reduced by eliminating potential infectious foci [9, 10]. It has been known for decades that attention should be centered on the presence of pre-operative dental infections [11]. Several studies have already described that oral health is an important influential factor in perioperative infections and can be the cause of a PJI in up to 15% of cases [12]. However, there are currently no clear recommendations or guidelines regarding the implementation of standardized, preoperative dental screening or care concepts. Some dentists already perform such an oral screening in cooperation with medical colleagues, but attest to the necessity of long-term follow-up examinations with regard to a possible postoperative benefit [10, 12]. Due to the special status of oral health in most health care systems, standardized preoperative oral screening could be an important factor in the prevention of PJI. It might help reduce the amount of PJIs in view of the increasing number of arthroplasty implantations. In addition, the routine dental examination, which is usually carried out every six months, would not require any additional economic expenditure. In these cases, the interdisciplinary communication between the dentist and the orthopaedist appears relevant, especially because limitations in this context were already reported [13].

Recent research on the necessity of an antibiotic prophylaxis for dental interventions after implantation of endoprostheses, which found no benefit of this prophylaxis, indicated that preoperative dental screening was recommendable to influence the occurrence of PJI [14–17]. However, deficits in the often-demanded establishment of interdisciplinary cooperation between orthopedists and dentists have been found mainly in the practical implementation of the procedures [13]. One university-based concept of dental referral has already been introduced, and it showed a high amount of oral foci in patients prior to joint replacement, but this concept was very time-consuming and extensive, making its general practicability, especially outside big cities, questionable; moreover, an effect on the occurrence of PJI could not be proven with this concept [18]. Therefore, a dental screening strategy prior to endoprosthesis implantation, which is practicable, i.e., non-time-consuming, easily applied, and transferable with broad acceptance at all endoprosthesis centers, as well as effective at reducing PJI, appears important and a reasonable challenge.

Following these thoughts, the objective of the present study was to reveal whether a preoperative dental screening with practical capability would reduce the prevalence of early PJI. Thereby, practical capability should be ensured by a form-based referral to family dentists prior to surgery, which should enable an easy, non-time-consuming, and cost-effective approach with broad acceptance for patients, orthopedists, and dentists. The underlying working hypothesis was that the implementation of a standardized preoperative dental screening with need-based dental intervention reduces the prevalence of PJI.

## Materials and methods

This current observational study was designed to compare the rates of early PJI between two groups of patients. The conceptualization of the study, data curation, and analysis were performed within a cooperation project. This cooperation included the Department of Oral and Maxillofacial Surgery, University of Leipzig, Germany, the Specialized Clinic for Orthopedics, Mediclin Waldkrankenhaus Bad Düben, Germany (SCBD), the Department of Cariology, Endodontology and Periodontology, University of Leipzig, Germany, and the Department of Orthopaedics, Trauma and Plastic Surgery, University Hospital Leipzig, Germany. The study has been reviewed and approved by the local ethics committee (No.: 116/20-ek). All study procedures were performed in full accordance with the declaration of Helsinki. All participants were informed verbally and in writing about the study and gave their written informed consent for participation.

### Study participants

The whole study period of the current investigation was between September 2018 and September 2022. Thereby, a test group (09/2020–09/2022) and a comparison group (retrospectively assessed from the period between 09/2018 and 08/2020) were recruited. Patients, who attended the SCBD for insertion of an endoprosthesis (hip, knee, or sledge) and who met the inclusion and exclusion criteria (see below), were consecutively asked for their participation and informed about the study. Where applicable, they gave their written informed consent. Two groups were formed.

#### Test group

A test group, which received a dental referral with the request to complete a standardized form, was recruited between September 2020 and September 2022. The following inclusion criteria for participating were defined:

- Implantation of an endoprosthesis within the study period
- Elective implantation (no acute surgery, e.g., trauma) of either hip, knee, or sledge prosthesis
- Primary implantation of the endoprosthesis
- Age of at least 18 years

Additionally, the following exclusion criteria were defined:

- Revision of endoprosthesis
- Postoperative complications, which required stationary therapy in another clinic
- Surgical wound revision without germ verification
- Acute endoprosthesis insertion, trauma, secondary surgery (revision of an existing endoprosthesis)
- Poor general health status, making a dental referral/examination impossible

Further inclusion and exclusion criteria did not exist.

#### Comparison group

To compare the findings of the test group, in which the dental referral concept was performed, a comparison group was assessed. This group attended the SCBD prior to the onset of the dental referral concept. Those patients received an endoprosthesis implantation between September 2018 and August 2020.

Inclusion and exclusion criteria for participation in the current study were equal between both groups.

### Data curation

**Fig. 1:**
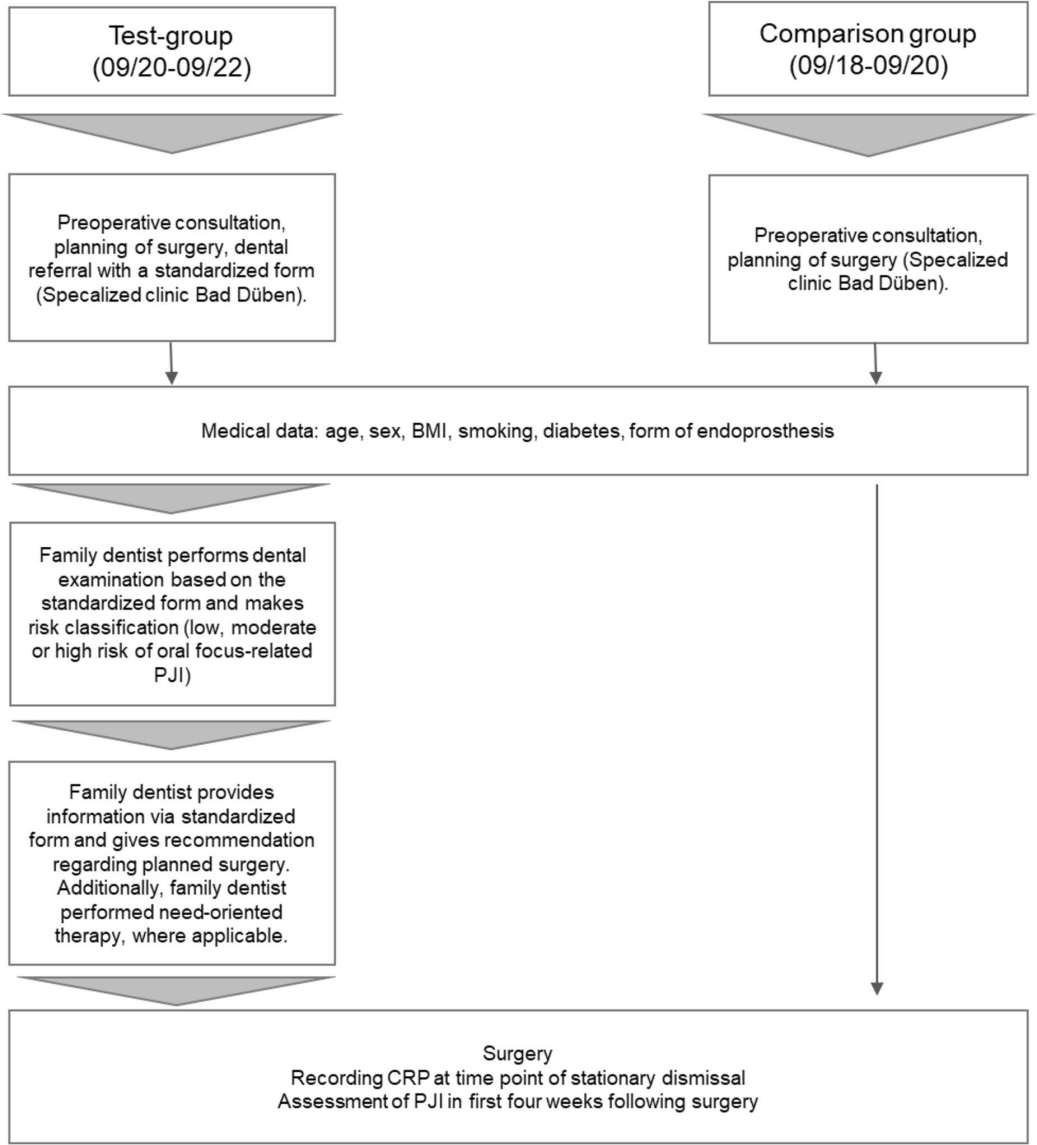
Data curation in the current study

The data curation included medical findings and the dental report and was performed according to the group allocation (dental report only in test group, medical findings were equal between groups) as displayed in Fig. 1.

#### Medical data

For the whole study cohort, several general or medical data were assessed, respectively.

The following information was recorded from the medical records of the participants: age, sex, smoking habits (smoker or non-smoker), presence of diabetes mellitus type II (yes or no), and body mass index (BMI). In the postoperative follow-up, two relevant pieces of information were recorded: the C-reactive protein (CRP) on the day of stationary dismissal and the occurrence of PJI during the first four weeks after surgery, which corresponds to an early PJI. Thereby, the presence of a PJI was based on the definition of the Musculoskeletal Infection Society (MSIS) in 2018, which included clinical, laboratory, and intra-operative findings, accordingly [19]. All patients attended the SCBD in case of infectious complications in the first month after surgery. This allowed reliable recording of any PJI within this period. This was possible because all rehabilitation clinics were attached to the SCBD and a presentation of the patients in the event of signs of infection was mandatory. As this was ensured only in the first four weeks after surgery, the period for PJI was limited to this first month although the definition indicates a period of three months for PJI.

#### Dental screening and referral concept

**Fig. 2:**
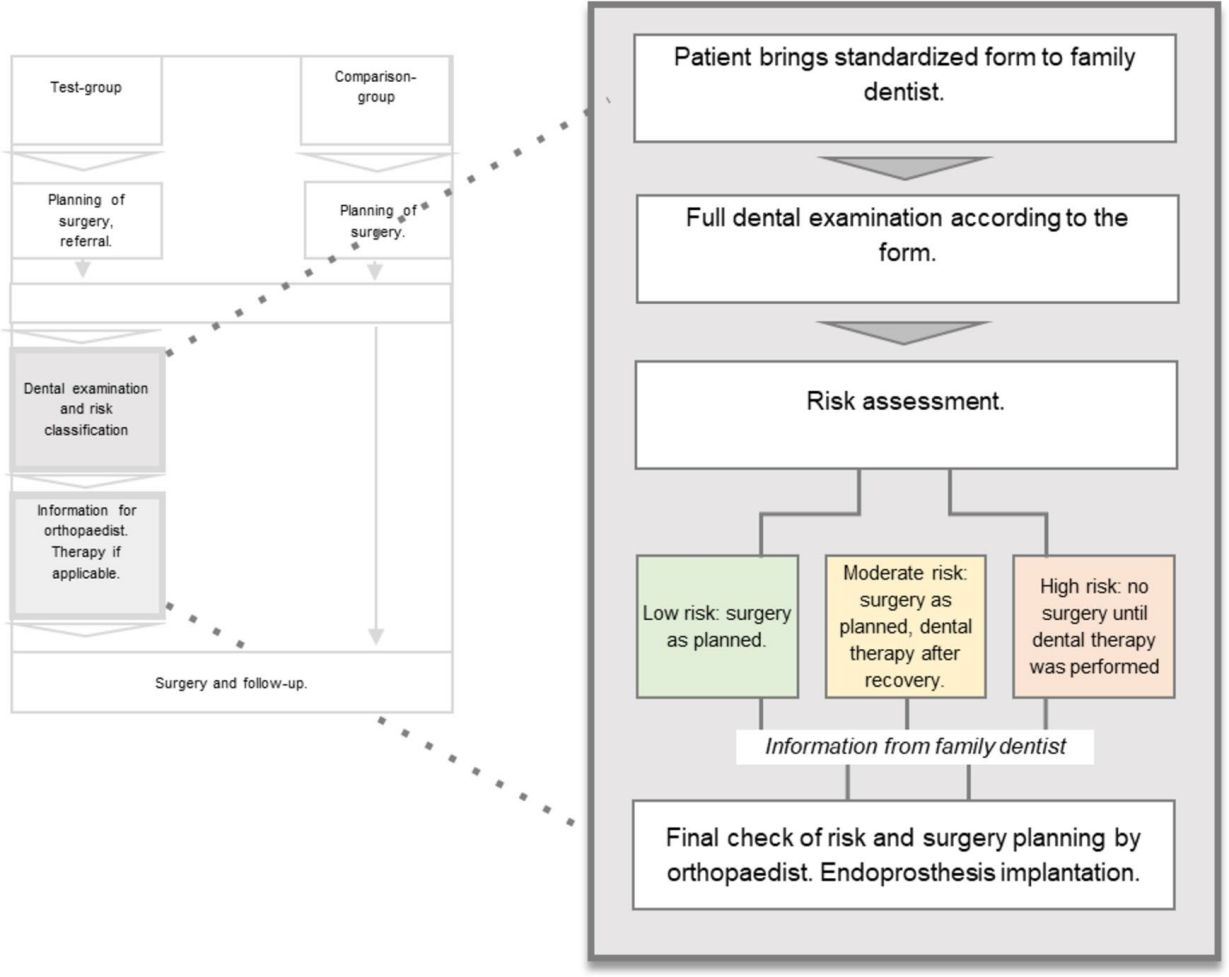
Procedure for risk assessment and evaluation of the family dentist

Members of the test group were referred to their family dentists prior to surgery (Fig. 2). Following the regular pre-operative consultation for planning an endoprosthesis in the SCBD, the patients received a standardized dental consultation form (supplementary Fig. 1) and were instructed to visit their family dentists. This form included information on the planned endoprosthesis implantation, the time point of the planned surgery, and the instruction to examine the oral cavity regarding potential oral foci of infection. The attending dentists were asked to classify the patient’s risk of the presence of an oral infection that might cause the dissemination of bacteria, which could be a source of peri-prosthetic colonization. This was classified into low (no further need of dental therapy, surgery can be performed as planned), moderate (further need of dental therapy, surgery can be performed as planned and dental therapy can be done afterward), or high (risk of oral disease-related PJI, mandatory need for dental rehabilitation prior to EP surgery). In the case of high risk, no endoprosthesis implantation was performed until need-oriented dental therapy was finished. This concept was based on a previous university-based study [18] but was simplified and adopted for use in practice. In brief, the previous concept included the dental referral of each patient prior to surgery from the university department of orthopedics to the university dental clinic. Thereby, specialized dentists performed a full dental examination and organized need-oriented therapy based on the aforementioned risk classification system by the respective family dentist [18]. In the current study, the referral was made directly to the family dentist, without a specialized control instance. Furthermore, a clear and simple consultation form was used to ensure appropriate information exchange in the current investigation. The form was mandatory for endoprosthesis surgery, which would ensure that all patients had visited their family dentists for check-ups.

### Statistical analysis

The statistical analysis was performed using SPSS for Windows, version 24.0 (SPSS Inc., US).

Kolmogorov–Smirnov test was applied to check the metric variables regarding their normal distribution. In comparing two independent, non-normal distributed samples, Mann–Whitney *U* test was used. More than two independent, non-normal distributed samples were compared using Kruskal–Wallis test. Categorical and nominal data were analyzed by chi-square or Fisher’s test, respectively.

In a subsequent univariate regression analysis, PJI was set as the dependent variable and was examined regarding the influential factors of age, BMI, gender, diabetes (yes vs. no), smoking, and dental referral (test group vs. comparison group). The significant or nearly significant findings in the univariate regression analysis were later included in a multiple logistic regression analysis. For all tests, the significance level was set at *p* < 0.05.

## Results

### Participants

In total, 2560 individuals were included in the current study. Of these, 1227 patients were in the test group and thus were referred to their family dentist prior to surgery. The comparison group consisted of 1333 individuals who did not receive a dental referral.

There were no significant differences in age, sex, diabetes, smoking, or BMI between the study groups (*p* > 0.05, Table 1). The type of inserted endoprosthesis differed slightly but significantly between the two groups (*p* < 0.01, Table 1).

**Table 1:** Participant characteristics, medical data, and PJI between test group and comparison group

	Test group (*n* = 1227)	Comparison group (*n* = 1333)	*p* value
Age in years (mv ± sd)	69.55 ± 9.36	69.77 ± 9.76	0.45
Sex (male in %, [*n*])	40.9% [502]	40.7% [542]	0.90
Form of endo-prosthesis % [*n*]			
Hip-TEP	52.9% [649]	50.5% [673]	** < 0.01**
Knee-TEP	40.7% [500]	39.5% [526]	
Sledge	6.4% [78]	10.1% [134]	
Diabetes mellitus II (yes in % [*n*])	17.1% [210]	17.0% [226]	0.92
Smoking (yes in % [*n*])	10.5% [129]	10.6% [141]	0.99
BMI (mv ± sd)	30.01 ± 5.75	29.81 ± 5.36	0.55
CRP (mv ± sd)	80.9 ± 44.47	87.93 ± 46.24	** < 0.01**
PJI (yes in % [*n*])	0.8% [10]	1.8% [24]	**0.04**
Risk assessment by family dentist % [*n*]			
Low	79.6% [977]		
Moderate	18.9% [232]		
High	1.5% [18]		

Significant findings (*p* < 0.05) are highlighted in bold

### PJI

In the test group (with dental referral), 10 patients developed PJI during the observation period. There were 24 early PJIs in the control group, which was significantly more than in the test group (0.8% vs. 1.8%, *p* = 0.04). Patients in the test group also had lower CRP levels on the day of hospital discharge (80.9 ± 44.47 vs. 87.93 ± 46.24, *p* < 0.01).

### Univariate analysis

Univariate analysis was performed with PJI as the dependent variable. Sex (OR: 2.37, CI_95_ 1.18–4.76, *p* = 0.02), BMI (OR: 1.07, CI_95_ 1.02–1.13, *p* = 0.01), and dental referral (test group) (OR: 0.45, CI_95_ 0.21–0.94, *p* = 0.03) showed significant results. There was also a trend for age (OR:1.03, CI_95_ 0.99–1.07, *p* = 0.08; Table 2). Therefore, these four parameters were included in the multiple logistic regression.

**Table 2:** Univariate analysis of potential predictors of PJI

	OR	CI_95_ lower	CI_95_ upper	*p* value
Age	1.03	0.99	1.07	0.08
Sex	2.37	1.18	4.76	**0.02**
BMI	1.07	1.02	1.13	**0.01**
Diabetes	1.27	0.55	2.93	0.60
Smoking	0.82	0.25	2.70	0.74
Dental referral (test group)	0.45	0.21	0.94	**0.03**

Significant findings (*p* < 0.05) are highlighted in bold

### Multiple logistic regression

Dental referral was a strong predictor of less PJI, with an OR of 0.43 (CI_95_ 0.205–0.917, *p* = 0.03; Table 3). On the other hand, male sex was strongly associated with more PJI (OR: 2.68, CI_95_ 1.32–5.42, *p* = 0.01). Age (OR: 1.056, CI_95_ 1.014–1.099, *p* = 0.01) and BMI (OR: 1.11, CI_95_ 1.05–1.170, *p* < 0.01) had little effect on PJI.

**Table 3:** Multiple logistic regression of potential predictors of PJI

	PJI	
	OR [CI_95_]	*p* value
Age	1.06 [1.01–1.10]	**0.01**
BMI	1.11 [1.05–1.17]	** < 0.01**
Sex	2.68 [1.32–5.42]	**0.01**
Dental referral (test group)	0.43 [0.21–0.92]	**0.03**

Significant findings (*p* < 0.05) are highlighted in bold

## Discussion

The current study showed that a dental referral using a standardized resulted in a remarkable reduction in early PJI. Statistical analysis confirmed the dental referral as an independent predictor of less PJI. Moreover, age, gender, and BMI were found to be influential factors in the occurrence of PJI.

PJI is a major challenge for the orthopedic community, as it is a very serious complication of endoprostheses [20]. Although PJI is rare, the resulting morbidity, impairment of quality of life, and associated costs are significant [6, 7]. Therefore, the current study addressed this highly relevant issue and aimed to assess whether a dental referral concept would lead to a reduction in the prevalence of early PJI. In general, the risk prediction of PJI has been extensively discussed in the literature. A recent prediction model for early PJI, which was applied in Sweden and Denmark, consisted of the parameters diagnosis leading to endoprosthesis insertion, BMI, American Society for Anesthesiologists (ASA) class, sex, age, and the presence of five defined comorbidities [21]. A large-scale German study found BMI to be an important risk factor for PJI after hip replacement [22]. Additionally, a retrospective evaluation of 36,494 patients with primary total hip replacement confirmed three major risk factors for PJI, i.e., obesity, coronary artery disease, and pulmonary hypertension [23]. Similarly, a study from New Zealand, which included more than 20,000 individuals after hip and knee replacement found male gender and BMI to be highly important risk factors for PJI [24]. Taken together, the increased risk of PJI associated with BMI, age, and male gender, which was found in the current study, appears to be consistent with the literature. Interestingly, in the current study, BMI was found to be only slightly related to PJI, as the OR was 1.11 in the multivariate analysis. On the other hand, male gender was a strong predictor of PJI in the current study, which is not strongly supported by the literature; however, due to the higher susceptibility of men to obesity [25], cardiovascular disease [26], and poorer health behavior [27], which are also potential risk factors for PJI, the findings seem somewhat plausible.

Those risk factors are difficult to modify and control, making short and practical interventions difficult. None of the reviewed previous studies, which focused on the detection of potential risk factors for PJI, addressed dental care as a potentially relevant factor. A recent review article by Young et al. (2021) concluded that oral health plays a small but relevant role in the occurrence of PJI [16]. The oral cavity was found to be one source of hematogenous PJI [28]. Consequently, several studies have focused on the potential impact of oral diseases or oral care on PJI, respectively. For example, the effects of tooth extraction, implant placement, caries, or dental scaling have been investigated in relation to PJI [29, 30]. Given that antibiotic prophylaxis after joint replacement does not reduce the risk of PJI [14, 17], the relevance of dental care prior to endoprosthetic implantation seems reasonable. This presumption is in line with recent literature [15]. However, the effect of a preoperative dental care concept has not yet been clarified yet. Additionally, an appropriate and practicable concept is still lacking. A previous study introduced a university-based concept, in which patients were referred from the orthopedic clinic to the department of cariology and periodontology for an oral examination and subsequently referred to their family dentist [18]. Although this previous study developed a risk classification concept, which was also used in the current study, the university-based concept was very comprehensive and cost-intensive, and the effect on PJI remained unclear. Therefore, the current study applied a practicable approach, in which patients were only referred to their family dentists and instructed to provide a report on a standardized form. Interestingly, this simple intervention reduced the prevalence of early PJI by more than 50% (see Table 1). Accordingly, the dental referral was an independent and reasonable predictor of reduced PJI. Therefore, the effect of a dental referral with a standardized form appears to be valid in this cohort and recommendable for general practice. As a side effect, the CRP was lower in the test group (dental referral group) on discharge from hospital. While a preoperative increase in CRP was reported to predict infections, the postoperative CRP during the first days after endoprosthesis was not a predictor of early PJI [31]. Accordingly, the finding of the current study regarding CRP is interesting but seems less clinically relevant.

A full discussion of the strengths and limitations of this investigation is required. In principle, this was the first large-scale study to confirm the benefit of preoperative dental referral when it comes to the prevalence of PJI using a clear and practicable concept. The groups were reasonable and balanced in terms of key demographic parameters. The dental referral concept is based on previous research in the field, where the risk classification system has been explained in detail [18]. However, the results of the current study must be interpreted with caution, as several important issues require recognition. Per definition, early PJI can occur within the first three months after endoprosthesis implantation [32]. While the current study design ensured a mandatory presentation of the patients in the SCBD within only the first four weeks after surgery, the current study´s findings are limited to this time. Future studies must confirm the current results by ensuring a longer post-operative observation period. The study design was retrospective in terms of groups, with the comparison group recruited first and the test group second. For a more robust conclusion, a parallel design would be needed. In addition, COVID-19 pandemic occurred during the study period, which might have influenced some of the findings (e.g., patient selection, no surgery for patients with comorbidities or poorer general health). Although patients in the test group were referred to their dentist with a standardized form, the type of therapy that each dentist provided was not checked. This is a limitation but also of potential interest. The effect on the prevalence of PJI was remarkable, although it was not checked as to whether the dentists really provided the respective need-oriented therapy. Thus, it can be assumed that dentists would have performed adequate dental therapy, eliminating potential oral foci with a risk of bacterial dissemination. However, this would require further examination. In particular, the appropriateness of the dental treatment must be questioned, as only 1.5% of the patients were classified as “high-risk,” whereas the university-based study found more than 30% at high risk [18]. However, this risk classification might have already been influenced by the dentists’ interventions. For example, the respective dentist might have extracted an inflamed tooth, resulting in a switch from high to moderate risk. In this context, it remains somewhat unclear whether the reduced prevalence of PJI was an effect of dental care or, rather, a kind of patient selection. It is known that, prior to endoprosthesis implantation, patients show a lack of oral health behavior and oral health awareness [33, 34]. Therefore, it could be conceivable that those patients went to another clinic for joint replacement. In any case, for endoprosthesis implantation as elective surgery, even an effect due to patient selection seems highly relevant.

In conclusion, the application of a structured, form-based, and consistent dental referral led to a reduction in the prevalence of PJI although the mechanisms of the effect remain unresolved. Further research is needed to prospectively validate the findings in a parallel design, including a review of the interventions that each dentist provided.

## Conclusion

Within the limitations of the current study, dental referral with a standardized form and respective risk classification can reduce the prevalence of early PJI. Accordingly, orthopedic surgeons and dentists should collaborate in this practical way to control the oral cavity as a risk factor for infectious complications in endoprostheses.

### Supplementary Information

Below is the link to the electronic supplementary material.

Supplementary file1 (PDF 136 KB)

## Data Availability

The datasets used and/or analyzed during the current study are available from the corresponding author on reasonable request. The data are not publically available because of the pseudonymization and data protection guidelines according to the ethics approval.
