# Supplementary material for: Preoperative dental screening can reduce periprosthetic infections of hip and knee endoprostheses in the first month after surgery: results of a cohort study
Source: Infection. 2023 Dec 7;52(2):535–43. doi: 10.1007/s15010-023-02128-2 (PMC10954939; doi:10.1007/s15010-023-02128-2)
Supplement: Supplementary file 1 — Supplementary file1 (PDF 136 KB) [file 15010_2023_2128_MOESM1_ESM.pdf]

Dental Screening

„Examination on the influence of oral health on early periprosthetic joint infections"

Dear patient, **Date:**

please hand this letter to your dentist.
Bring the completed form with you to your next appointment with the surgical team.

Dear colleague,

Mr./Ms.…………………………………..
Is scheduled for an endoprosthesis insertion.
We therefore kindly ask you to complete this screening form, in order to exclude oral diseases that may pose a risk for endoprosthesis infection.
Please pass on the following information to us:

	**yes**	**no**
Is periodontal treatment needed?	☐	☐
Is treatment of carious lesions needed?	☐	☐
Do any of the following oral diseases exist:	☐	☐
• carious destroyed teeth	☐	☐
• strong need for periodontal treatment (Periodontal Screening Index (PSI) 3-4 in more than 2 sextants, suppuration, perio-endo-lesions)	☐	☐
• endodontically and/or peri-apically infected teeth	☐	☐
• inflammatory process in the jawbone	☐	☐
• partially retained or impacted wisdom teeth with formation of peri-coronal inflammation or cyst	☐	☐
• disease of masticatory muscles / jaw joint	☐	☐

The patient`s risk of an infectious complication of the endoprosthesis with oral cause is

☐ Low ☐ Moderate ☐ High

The insertion of an endoprosthesis

☐ Can be performed as planned
☐ Can be performed as planned, but dental therapy should be scheduled for afterwards
☐ Should not be performed before dental treatment has been completed

Date:…………………………… Signature:…………………………………………..
